# Detection of missed deaths in cancer registry data to reduce bias in long-term survival estimation

**DOI:** 10.3389/fonc.2023.1088657

**Published:** 2023-03-09

**Authors:** Stefan Dahm, Benjamin Barnes, Klaus Kraywinkel

**Affiliations:** German Center for Cancer Registry Data, Department for Health Monitoring, Robert Koch-Institute, Berlin, Germany

**Keywords:** cancer registry data, missed deaths, long-term survival, classification algorithm, relative survival

## Abstract

**Background:**

Population-based cancer survival estimates can provide insight into the real-world impacts of healthcare interventions and preventive services. However, estimation of survival rates obtained from population-based cancer registries can be biased due to missed incidence or incomplete vital status data. Long-term survival estimates in particular are prone to overestimation, since the proportion of deaths that are missed, for example through unregistered emigration, increases with follow-up time. This also applies to registry-based long-term prevalence estimates. The aim of this report is to introduce a method to detect missed deaths within cancer registry data such that long-term survival of cancer patients does not exceed survival in the general population.

**Methods:**

We analyzed data from 15 German epidemiologic cancer registries covering the years 1970-2016 and from Surveillance, Epidemiology, and End Results (SEER)-18 registries covering 1975-2015. The method is based on comparing survival times until exit (death or follow-up end) and ages at exit between deceased patients and surviving patients, stratified by diagnosis group, sex, age group and stage. Deceased patients with both follow-up time and age at exit in the highest percentile were regarded as outliers and used to fit a logistic regression. The regression was then used to classify each surviving patient as a survivor or a missed death. The procedure was repeated for lower percentile thresholds regarding deceased persons until long-term survival rates no longer exceeded the survival rates in the general population.

**Results:**

For the German cancer registry data, 0.9% of total deaths were classified as having been missed. Excluding these missed deaths reduced 20-year relative survival estimates for all cancers combined from 140% to 51%. For the whites in SEER data, classified missed deaths amounted to 0.02% of total deaths, resulting in 0.4 percent points lower 20-year relative survival rate for all cancers combined.

**Conclusion:**

The method described here classified a relatively small proportion of missed deaths yet reduced long-term survival estimates to more plausible levels. The effects of missed deaths should be considered when calculating long-term survival or prevalence estimates.

## 1 Introduction

The survival of cancer patients documented by population-based cancer registries can be used to analyze the real-world impacts of healthcare interventions, such as new treatment methods or changes in preventive measures. However, the estimation of survival rates can be biased by missing or invalid data. In Germany, population-based cancer registries collect information about cancer type, date of diagnosis and vital status of patients within the borders of federal states. Vital status is obtained with passive follow-up through linkage with the death notifications of the population registries. Passive tracking of vital status can be affected by several types of error. First, incorrect patient data (misspelling of names or transposed digits in birth dates) can prevent the correct link to the death notification. Linkage may also fail when the incidence notification is delayed and the survival time is short. Finally, migration between federal states may cause problems if registries are only able to link data to population registries in their own respective federal state. In all these cases the patients are assumed to be alive by the registries at the end of follow-up (unless the date of emigration is known and can be used to censor follow-up), resulting in seemingly immortal patients. The proportion of such missed deaths increases with follow-up time as other patients successively die. This leads to an ever-greater overestimation of survival rates with increasing follow-up time. The problem is most pronounced in older age groups, in which survival times are shortest.

The impact of missed deaths on survival estimation was found to be stronger for relative survival than for absolute survival (1). Relative survival is calculated by the quotient of the survival rate in the patient group and the survival rate in the general population matched by age, sex and calendar year. A high proportion of missed deaths can lead to a higher survival rate in the patient group than in the general population, resulting in relative survival exceeding 100%. Bias related to missed deaths is not only relevant for the calculation of long-term survival rates, but also for estimates of long-term or lifetime prevalence.

A limited number of studies have investigated the problem of missed deaths in population-based cancer registry data. Pinheiro et al. (2) estimated the proportion of missed deaths in the SEER data based on the proportion of registered long-term survivors with high-grade lethal cancer. In that study, missed deaths were defined as persons with survival time of more than 5 years according to the data, but who were contacted for the last time between 0 and 5 years after diagnosis. Sriamporn et al. (3) described a loss-adjusted approach to estimate dates of death, examining cancer cases of the cervix uteri in Thailand. For patients whose vital status could not be determined at follow-up end, annual mortality probabilities were estimated with logistic regression. This regression was first fitted to data from patients with similar characteristics with known survival times. Okuyama et al. (4) conducted a simulation study to investigate the bias in absolute 5-year survival rates that results from missed deaths. At 5% loss to follow-up, lung cancer survival was overestimated by 4% and breast cancer survival was overestimated by 1%. In general, survival estimates for cancers with poor prognosis showed the highest bias due to missed deaths.

The aim of this report is to introduce an algorithm to detect missed deaths in population-based cancer registry data such that long-term survival of cancer patients does not exceed survival in the general population. In order to estimate long-term cancer prevalence according to the method developed by (5), we analyzed survival times up to 35 years. The method is applied to German cancer registry data and to SEER data and tested by simulations. The algorithm will be implemented in the R package ‘misseddeaths’ to be published on GitHub.

## 2 Materials and methods

### 2.1 Cancer registry data

This study included data from nearly 5.0 million cancer patients diagnosed at age 15 years and older registered in one of fifteen German population-based cancer registries between 1970 and 2016. Only the cancer registry of Saarland has been operating continuously since 1970. In the other federal states, registration began in 1990 or later (**Table 1**). From the federal states in eastern Germany (Berlin, Brandenburg, Mecklenburg-West Pomerania, Saxony-Anhalt, Saxony and Thuringia), data was available only between 1995 and 2015. For survival analysis, the follow-up time for patients in these eastern German registries was censored on 31 December 2015 and for patients in other registries on 31 December 2016. All data were transmitted from the state cancer registries to the German Centre for Cancer Registry Data at the Robert Koch Institute according to federal law.

**Table 1:** German data: Analysis period, number of patients, percent deaths, median age at diagnosis and 10-year relative survival for all cancers combined by German federal state.

Registry	analysis period	Number of patients	deaths [%]	median age at diagnosis [years]	10-year relative survival rate [%]
**Schleswig-Holstein**	1999-2016	253,106	48.4	67.8	64.3
**Hamburg**	1990-2016	200,092	60.2	67.9	59.4
**Lower Saxony**	2003-2016	572,850	46.9	68.7	59.6
**Bremen**	1998-2016	61,235	56.2	68.8	57.9
**NRW without Muenster**	2007-2016	777,884	41.0	68.6	60.1
**Muenster**	1990-2016	305,622	60.1	68.3	59.7
**Rhineland-Palatinate**	1998-2016	336,660	50.0	68.0	63.7
**Bavaria**	2002-2016	815,680	43.3	67.8	67.5
**Saarland**	1970-2016	211,560	71.9	67.4	57.4
**Berlin**	1995-2015	237,535	47.7	66.2	68.9
**Brandenburg**	1995-2015	233,471	54.6	67.2	55.7
**Meckl-West. Pom.**	1995-2015	161,159	55.7	67.2	54.7
**Saxony**	1995-2015	428,577	58.4	69.1	53.9
**Saxony-Anhalt**	1995-2015	199,893	54.9	67.5	54.1
**Thuringia**	1995-2015	202,312	54.3	67.8	56.4
**Total**	**1970-2016**	4,997,636	50.5	68.0	61.2

In order to evaluate the proposed method, we additionally analyzed SEER Research Data (6) from approximately 6.4 million cancer cases diagnosed between 1975 and 2015 (**Table 2**). SEER data were collected from 18 population-based cancer registries, with 9 registries providing data since 1975. The data sets of the other registries begin between 1992 and 2000. The analysis was restricted to whites, as this is the ethnic group in the USA with the lowest proportion of missed deaths, estimated to be below 1% (2, 7).

**Table 2:** SEER data: Analysis periods, number of patients, percent deaths, median age at diagnosis and 10-year relative survival for all cancers combined^1^ by SEER registry (race=white).

Registry	analysis period	Number of patients	deaths [%]	median age at diagnosis [years]	10-year relative survival rate [%]
**Alaska Natives**	1992-2015	0	0	-	-
**Atlanta (Metropolitan)**	1975-2015	218,522	60.7	64	67.2
**California excluding SF/SJM/LA**	2000-2015	1,049,953	48.5	67	61.3
**Connecticut**	1975-2015	553,988	68.5	68	65.5
**Detroit (Metropolitan)**	1975-2015	522,627	70.3	67	61.2
**Greater Georgia**	2000-2015	305,515	49.8	66	55.1
**Hawaii**	1975-2015	53,494	61.4	65	65.7
**Iowa**	1975-2015	500,009	71.5	69	60.0
**Kentucky**	2000-2015	315,889	53.1	66	52.5
**Los Angeles**	1992-2015	573,646	56.7	67	62.6
**Louisiana**	2000-2015	224,326	51.0	66	56.5
**New Jersey**	2000-2015	571,668	47.0	67	65.2
**New Mexico**	1975-2015	205,668	65.2	67	59.1
**Rural Georgia**	1992-2015	8,778	56.5	67	58.5
**San Francisco-Oakland SMSA**	1975-2015	465,880	68.2	67	68.8
**San Jose-Monterey**	1992-2015	159,557	52.8	66	69.1
**Seattle (Puget Sound)**	1975-2015	539,847	63.6	66	67.4
**Utah**	1975-2015	210,203	58.4	66	70.4
**Total**	**1975-2015**	6,479,570	58.9	67	62.0

For German data, tumors were classified according to Union for International Cancer Control (UICC) stage. For SEER data, tumors were classified by historic stage A, distinguishing between local, regional and distant tumors. Prostate cancer is classified by historic stage A into only two stages (“localized/regional” and “distant”). Since historic stage A is only available until the year 2015, we restricted the analysis of SEER data to the time period from 1975 to 2015. However, historical stage A was not available for all diagnoses throughout the entire period. For example, this stage information has been reported for lung cancer (C34) only since 1988 and for prostate cancer (C61) since 1995. For several diagnoses within oral cavity, pharynx and larynx localization group (C00-C14, C30-C32) and for Vagina (C52), historical stage A were reported only until 2003.

Generally, cases registered using information only from death certificates (DCO cases) or autopsy reports were excluded from the analysis. Cases with missing stage information were assigned to an extra category and included. In case of multiple primary cancers, the analysis was restricted to the survival time after the diagnosis of the last primary cancer.

### 2.2 Detection of missed deaths

**Figure 1:**
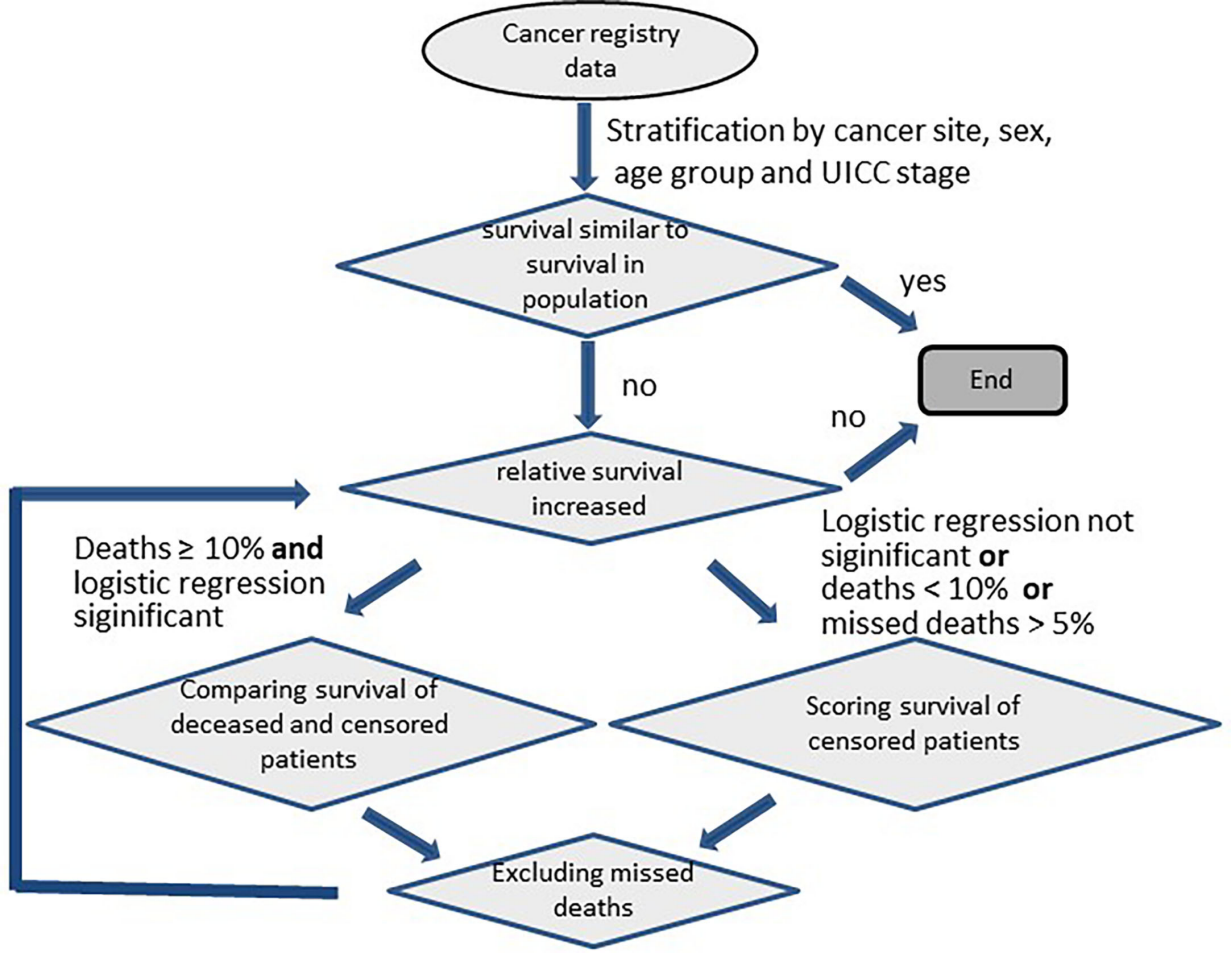
Analysis scheme for detection of missed deaths in cancer registry data.


We looked for missed deaths if the observed survival rate of cancer patients exceeded that of the corresponding overall population at the end of follow-up (**Figure 1**). To detect missed deaths, we compared ages and survival times of patients with documented dates of death with the corresponding data of patients documented as being alive at the end of follow-up. The maximum age and the maximum follow-up time of deceased persons should be greater than those values among persons alive at the end of follow-up, since the observation time of surviving persons is censored. For example, in SEER data the oldest deceased person with lung cancer was 108 years old at death, and the oldest living person with lung cancer at the end of follow-up (31 December 2015) was 101 years old. Both survived 18 years after diagnosis. In order to compare follow-up times and ages of deceased and surviving persons in as homogeneous groups as possible, the data were stratified according to diagnosis group, sex and age group (15-54, 55-74, 75 years and older at diagnosis). If there were at least 500 persons in each UICC stage (resp. each historic stage A for SEER data), the data were further stratified by stage with an additional stratum for cases with missing stage information. Within these strata, relative survival and 1-year conditional relative survival were estimated by period analysis. Relative survival was estimated over 35-years for persons younger than 55 years at diagnosis and over 30-years for older persons. Survival estimation was carried out by the R package ‘periodR’ (8) using the function ‘period’ with the Ederer II method (9) for calculating expected survival. The Ederer II method was used for estimating relative survival since the Ederer I method (10) or the Hakulinen method (11) tend to overestimate long term relative survival (12). Relative survival estimation was based on life tables covering the time period from 1993 to 2016 and the age range from 0 to 99 years (expected survival for ages over 99 years was assumed to equal the expected survival at 99 years of age). The life tables were stratified by sex, 1-year age group, and calendar year. For the analysis period we chose the last 5 years of follow-up (2011-2016 for German data and 2010-2015 for SEER data). Thus, survival estimates based on German data can include patients from diagnosis year 1976 onwards. We designated deceased persons with follow-up time and age at death in the 99.9th percentile as outliers and fitted a logistic regression on data from deceased persons with these outliers as events:

(1) $$logit(outlier)=\beta_{0}+\beta_{1}\cdot fu\_age+\beta_{2}\cdot fu\_time$$

*fu_age*: age at follow-up end

*fu_time*: follow-up time.

When the logistic regression did not converge, we lowered the percentile that defines deceased persons as outliers by 0.05 percentage points and carried out the regression again with the additional outliers. The procedure was repeated until the regression converged. If at least one of the coefficients *β*_1_ or *β*_2_ was significant at the 0.05 level, we used the regression to predict the probability of having missed the death of a person documented as being alive at the end of follow-up. Persons with probabilities ≥ 90% were assumed to have died. These persons were excluded from the data, and survival analyses were repeated. The search for missed deaths was iterated by successively lowering the percentile by 0.5 points each time until the survival rates of persons with cancer did not exceed those in the corresponding population.

**Figure 2:**
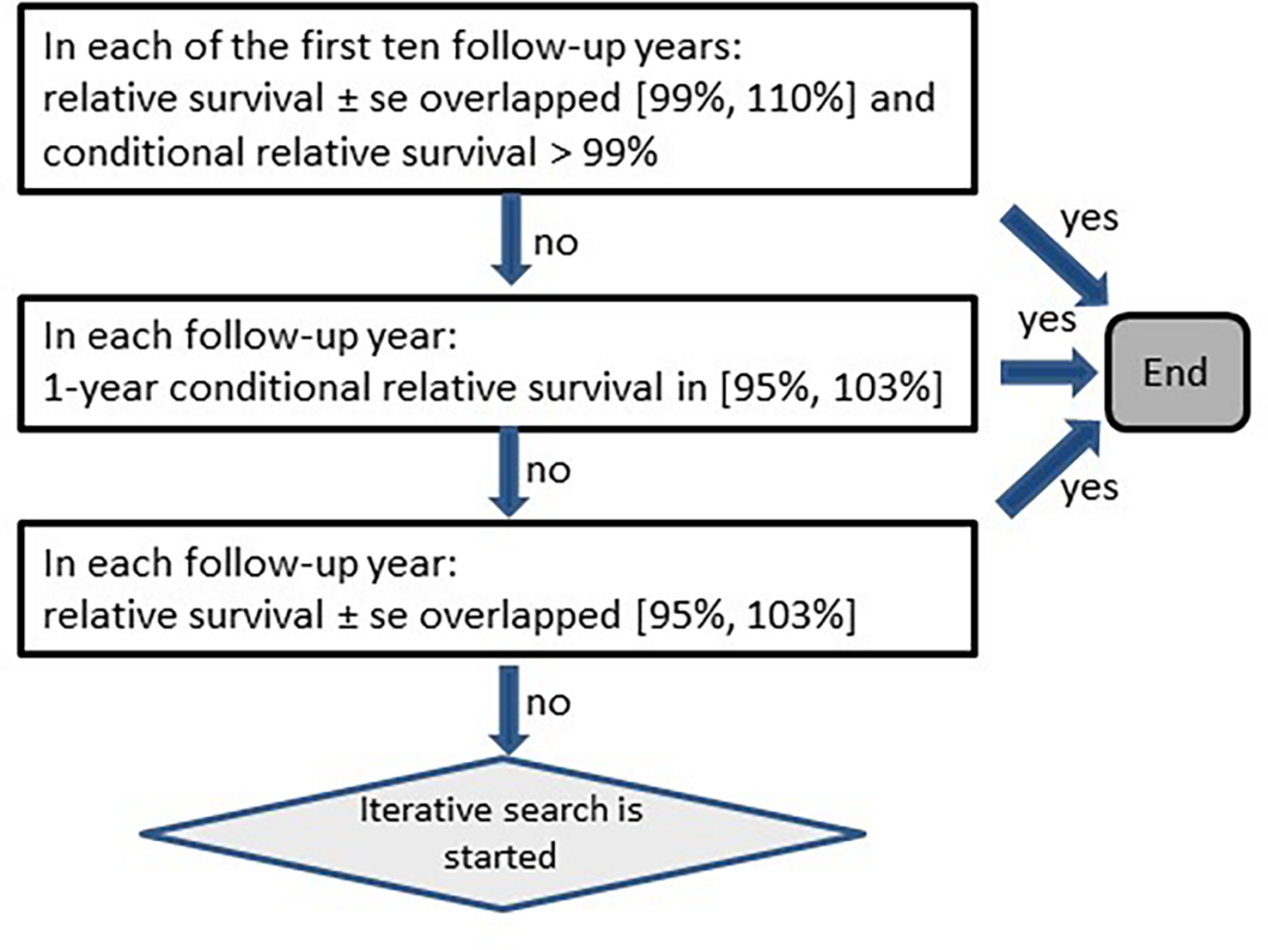
Scheme for testing on increasing relative survival (se, standard error).

**Figure 3:**
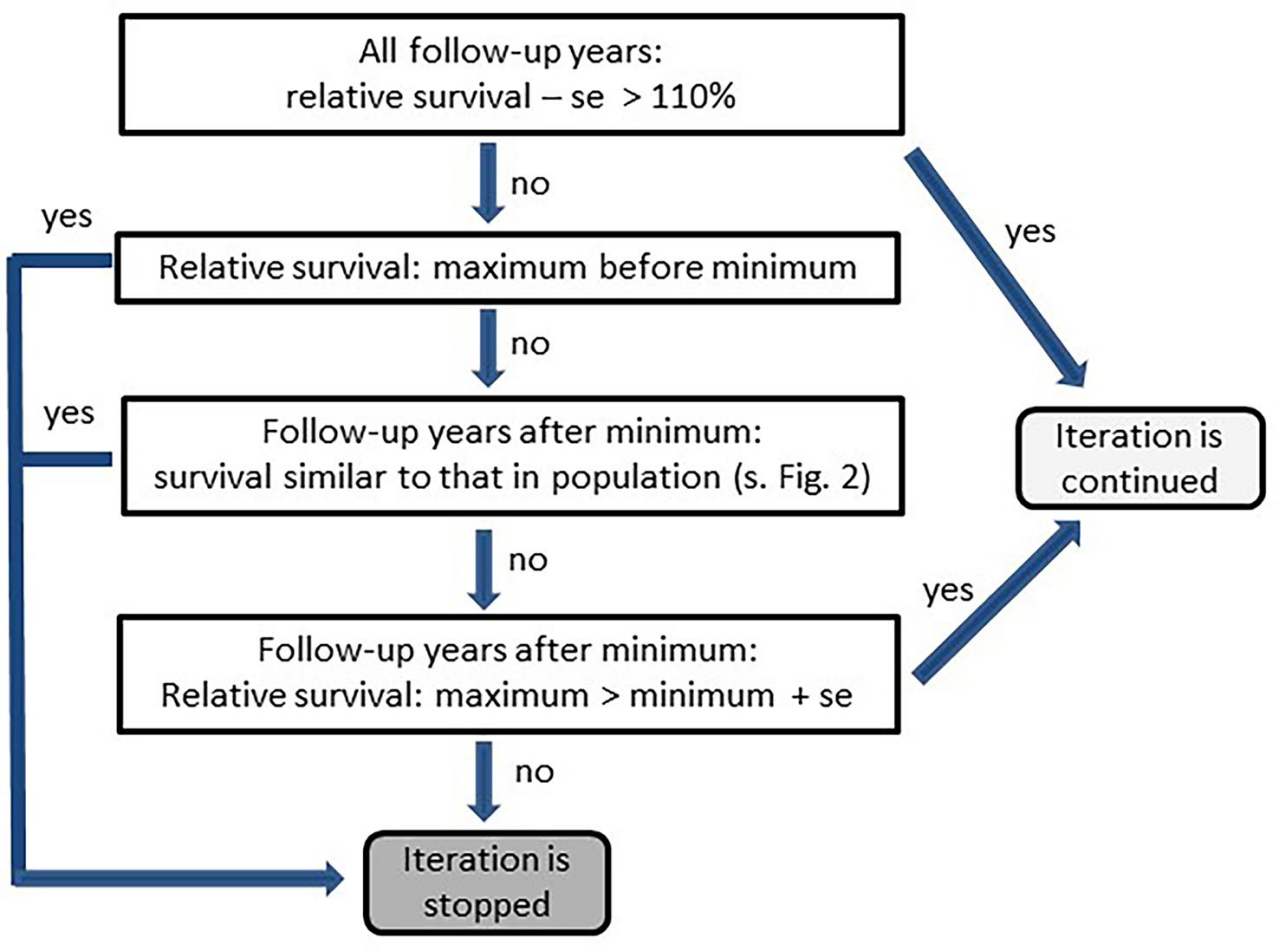
Scheme for testing differences between survival of cancer patients and survival of the corresponding population (se, standard error).

**Figure 4:**
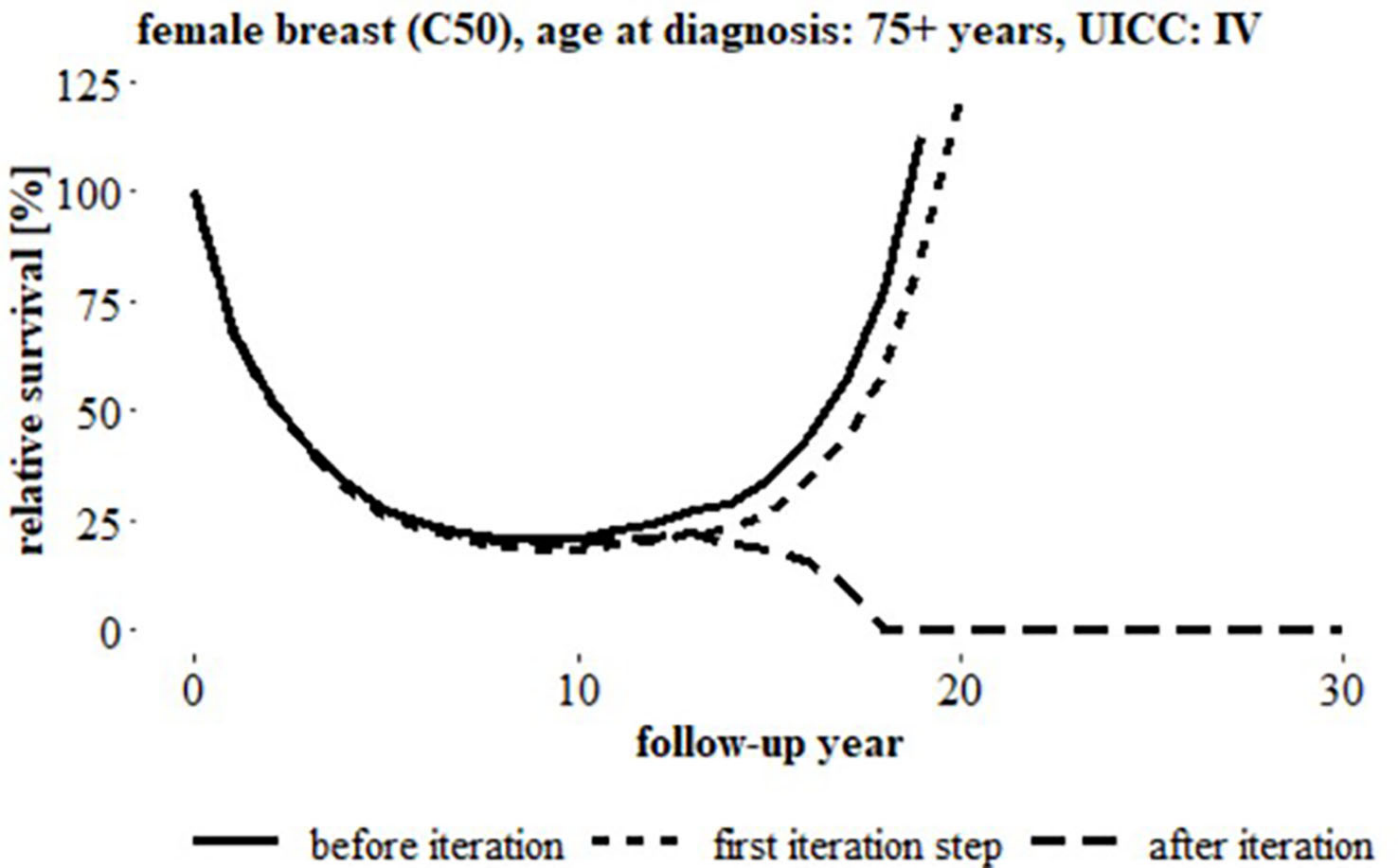
Relative survival over 30 follow-up years, breast cancer (C50), women, age at diagnosis: 75+ years, UICC status ‘IV’ (German data) before iteration, after first iteration step and after iteration, (uncorrected relative survival started to increase after 8 years follow-up).

Due to the large heterogeneity among cancers, the method was adapted to account for different disease courses. The adaptations presented here were tailored to reflect features of the SEER and German data. First, the algorithm had to determine for each stratum whether the survival rates of persons with cancer differed from those of the corresponding general population (**Figure 2**). When no differences were detected, the algorithm stopped without searching for missed deaths. Otherwise the classification of missed deaths was started in an iterative manner. Second, each iteration tested whether relative survival over the follow-up years increased after a minimum (**Figures 3**, **4**).

For strata with a small number of documented deaths, there was a high probability of observing only very few persons with high age at death and long follow-up time, making it difficult to compare the distributions of survival times and ages between deceased and surviving persons. This often led to a too high proportion of misclassified survivors. Therefore, for strata in which fewer than 10% of persons with cancer died, we classified missed deaths with a scoring algorithm using only the data of the surviving persons. The cutoff of 10% was introduced after examining the distribution of follow-up times and ages at follow-up end in the German data and in the SEER data. Of the 349 strata in the German data, 24 contained less than 10% deaths (**Table 3**). In these strata, there were on average 5.2 deceased patients with both age at death and survival time above the 99th percentile. In strata with 10% to 20% deaths resp. ≥ 20% deaths, the number of deaths with both age at death and survival time higher than the 99th percentile was on average 3 resp. nearly 5 times higher than in strata with less than 10% deaths. Of the 334 strata in the SEER data, 8 strata contained less than 10% deaths, each with on average 7.0 deaths with both age at death and survival time higher than the 99th percentile. In strata with 10% to 20% deaths resp. ≥ 20% deaths, there were on average 33.9 patients resp. 37.8 patients with both age at death and survival time higher than the 99th percentile.

**Table 3:** Characteristics of German and SEER cancer registry data strata* by proportion of patient mortality: number of patients, number of deaths and average number of deaths per stratum. Average number of deaths per stratum additionally presented according to percentile^#^ of age at death and survival time.

		German data				SEER data			
		Strata* with<10% patient mortality (N = 24)	Strata with ≥10% to<20% patient mortality (N = 25)	Strata with >20% patient mortality (N = 300)	All strata (N = 349)	Strata with<10% patient mortality (N = 8)	Strata with ≥10% to<20% patient mortality (N = 16)	Strata with >20% patient mortality (N = 310)	All strata (N = 334)
**Patients [N]**		415,071	535,256	4,047,309	**4,997,636**	235,371	891,624	5,352,575	**6479,570**
**Deaths [N]**		25,594	85,468	2,412,502	**2,523,564**	13,035	148,425	3,653,591	**3815,051**
**Average number of deaths per stratum**	**all**	1,066.4	3,418.7	8,041.7	**7,230.8**	1,629.4	9,276.6	11,785.8	**11,422.3**
	**≥ 95th percentile^#^**	30.5	93.8	134.2	**124.2**	46.9	249.9	222.4	**219.5**
	**≥ 99th percentile**	5.2	16.6	24.0	**22.1**	7.0	33.9	37.8	**36.9**
	**≥ 99.9th percentile**	0.7	1.4	1.9	**1.8**	0.1	2.2	2.7	**2.6**

*Stratification by diagnosis group, sex and age group and UICC in German data resp. historical stage in SEER data.

^#^Number of deaths with both age at death and follow-up time ≥ the resp. percentile.

The scoring algorithm was also applied when the proportion of deaths classified as missed deaths by the logistic regression was unrealistically high (> 5% of deaths) or if neither the coefficient for follow-up age nor the coefficient for follow-up time in the logistic regression (equation 1) was significant (**Figure 1**). For the scoring algorithm we used the Euclidean norm of the joint distribution of follow-up time (*fu_time*) in years and age at end of follow-up (*fu_age*) in years:


(2) $$score=\sqrt{fu\_age^{2}+fu\_time^{2}}$$

The data of surviving persons were ordered by this score. In the first iteration step, persons with scores in the 99.9th percentile were considered as having died and were excluded from the analysis. The iterations then continued as shown in **Figure 3** with the percentile for determining the cut-off score decreasing by 0.5 points each iteration. As with logistic regression, the iteration by scoring was stopped when the number of classified missed deaths exceeded 5% of the deaths.

For age standardization, weights from the international cancer survival standards (13) were used. Specifically, we applied the weights 0.19, 0.52, 0.29 to the age groups 15 to 54 years, 55 to 74 years and 75+ years, respectively.

The number of missed deaths for all cancers combined^1^ is the sum of missed deaths detected in the 23 individual diagnosis groups (**Table 4**).

**Table 4:** Number of patients, deaths and detected missed deaths and percentage of detected missed deaths among deceased patients by diagnosis group for German data and for SEER data.

	German data			SEER data		
Diagnosis group (ICD-10 Code)	patients [n]	deaths [n]	missed deaths [n (%)]	patients [n]	Deaths [n]	missed deaths [n (%)]
**Oral cavity, pharynx, larynx (C00-C14, C30-C32)**	184,991	103,012	738 (0.72)	214,194	132,105	33 (0.02)
**Esophagus, stomach (C15-C16)**	251,170	192,952	1,693 (0.88)	172,502	146,866	20 (0.01)
**Intestine (C17-C21, C26)**	713,309	391,996	4,141 (1.06)	723,196	478,874	16 (0)
**Liver, gallbladder, bile ducts (C22-C24)**	124,047	104,123	859 (0.82)	127,233	108,527	32 (0.03)
**Pancreas (C25)**	141,445	124,152	519 (0.42)	176,048	162,893	27 (0.02)
**Thorax (C33-C34, C37-C39, C45)**	574,300	476,417	2,154 (0.45)	933,743	829,143	6 (0)
**Malignant melanoma (C43)**	204,486	43,122	1,083 (2.51)	295,823	88,920	231 (0.26)
**Bones, soft tissues (C40, C41, C46-C49)**	44,174	22,591	235 (1.04)	82,679	51,305	33 (0.06)
**Breast (C50)**	791,557	227,326	2,220 (0.98)	957,499	373,349	19 (0.01)
**Vulva, vagina, uterine cervix (C51-C53)**	97,465	41,651	595 (1.43)	86,859	41,168	5 (0.01)
**Corpus uteri (C54 - C55)**	127,332	48,315	778 (1.61)	199,411	85,238	2 (0)
**Ovaries, other female genital organs (C56 - C58)**	93,349	59,790	489 (0.82)	117,272	80,887	63 (0.08)
**Male genital organs (C60, C62, C63)**	57,723	7,047	77 (1.09)	52,423	9,114	0 (0)
**Prostate (C61)**	625,176	198,413	2,836 (1.43)	875,432	366,773	0 (0)
**Urinary organs (C64 - C68, C74)**	351,936	186,809	1,995 (1.07)	481,187	269,131	33 (0.01)
**Central nervous system (C69 - C72)**	81,558	59,138	364 (0.62)	108,846	83,001	13 (0.02)
**Thyroid and other endocrine glands (C73, C75)**	67,122	10,790	202 (1.87)	146,517	18,413	134 (0.73)
**Hodgkin lymphoma (C81)**	24,179	5,274	45 (0.85)	40,800	13,236	0 (0)
**Non-Hodgkin Lymphoma (C82 - C88)**	161,772	74,906	579 (0.77)	273,704	158,368	0 (0)
**Multiple myeloma (C90)**	61,052	38,533	242 (0.63)	81,574	59,894	3 (0.01)
**Leukemia (C91 - C96)**	118,005	66,387	317 (0.48)	191,275	128,114	3 (0)
**Unspecified, ill-defined (C76, C80)**	101,488	87,380	1,119 (1.28)	141,353	131,124	23 (0.02)
all cancers combined^*^	**4,997,636**	**2,570,124**	**23,280 (0.91)**	6,479,570	3,816,443	696 (0.02)

*All cancers combined: All cancers excluding Non-Melanoma skin cancer (C00-C97 excl. C44).

### 2.3 Simulation

To evaluate the proposed method, we simulated missed deaths in the SEER data by randomly selecting deceased patients (1%, 2%, and 5%) in each registry. The vital status of patients in the sample was set to “alive” and the date of end of follow-up was set to December 31, 2015. Missed deaths previously detected by the algorithm were excluded. Simulations were repeated five times for each stratum (diagnosis group, sex, age group, and historical stage A). We recorded the proportions of detected missed deaths and analyzed the sensitivity (14) and the proportion of false positives, where the sensitivity is given by


(3) $$sensitivity=\frac{number\;of\;(simulated\text{ \& }detected)}{number\;of\;simulated}\cdot 100$$

Additionally, we estimated survival before and after applying the algorithm based on data sets with 5% simulated missed deaths. The numbers given in **Table 5** and **Table 6** are average values over the simulations.

**Table 5:** Number of patients, number of deaths, number of classified missed deaths, proportions [%] of detected missed deaths among the deaths, sensitivity and proportion [%] of false positives among the classified missed deaths by proportion of simulated missed deaths and diagnosis group, SEER data.

Diagnosis group	Patients [N]	Deaths [N]	Classified missed deaths			
			1% simulated	2% simulated	5% simulated	
			[N] (% of deaths, sensitivity %, false positive %)	[N] (% of deaths, sensitivity %, false positive %)	[N] (% of deaths, sensitivity %, false positive %)	
**Intestine (C17 - C21, C26)**	723,180	478,842	1,562 (0.3, 27, 16)	3,753 (0.8, 33, 15)	10,981 (2.3, 40, 13)	
**Pancreas (C25)**	176,021	162,839	679 (0.4, 38, 9)	1,450 (0.9, 42, 6)	3,980 (2.4, 47, 4)	
**Thorax (C33 - C34, C37 - C39, C45)**	933,737	829,131	2,961 (0.4, 32, 9)	6,168 (0.7, 35, 7)	17,386 (2.1, 40, 4)	
**Malignant Melanoma (C43)**	295,592	88,458	251 (0.3, 14, 52)	534 (0.6, 18, 41)	1,786 (2.0, 25, 39)	
**Breast (C50)**	950,965	370,028	611 (0.2, 14, 15)	1,351 (0.4, 16, 13)	6,355 (1.7, 27, 21)	
**Prostate (C61)**	875,432	366,773	1,177 (0.3, 27, 17)	3,275 (0.9, 33, 27)	9748 (2.7, 40, 25)	
**Total**	**3,954,927**	**2,296,071**	**7,241 (0.3, 27, 14)**	**16,531 (0.7, 31, 14)**	**50,236 (2.2, 38, 14)**	

**Table 6:** Age-standardized relative survival [%] by diagnosis group and follow-up year based on SEER data with 5% simulated missed deaths before and after applying the proposed method and SEER data without simulation (original estimate).

Diagnosis group	Data	follow-up year						
		5	10	15	20	25	30
**Intestine (C17 - C21, C26)**	**Before correction**	66.8	64.6	72.7	120.6	>200	>200
	**After correction**	66.7	63.7	65.0	59.4	52.1	45.4
	**SEER original estimate**	63.8	58.1	55.6	52.2	48.4	38.8
**Pancreas (C25)**	**Before correction**	15.9	17.2	30.6	107.8	>200	>200
	**After correction**	15.4	13.6	11.4	8.3	4.1	3.3
	**SEER original estimate**	9.9	6.9	5.6	4.4	2.8	1.9
**Thorax (C33 - C34, C37 - C39, C45)**	**Before correction**	25.0	22.8	31.6	90.7	>200	>200
	**After correction**	24.7	20.6	19.0	14.2	10.8	5.2
	**SEER original estimate**	19.6	13.6	10.2	7.6	6.2	4.2
**Malignant Melanoma (C43)**	**Before correction**	91.4	93.4	104.1	152.4	>200	>200
	**After correction**	91.3	92.6	95.6	85.6	71.0	55.6
	**SEER original estimate**	89.6	88.5	89.4	90.2	82.1	79.2
**Breast (C50)**	**Before correction**	92.3	90.5	94.4	120.8	>200	>200
	**After correction**	92.3	90.2	89.8	80.3	69.4	67.1
	**SEER original estimate**	90.9	86.7	83.4	79.5	75.0	59.3
**Prostate (C61)**	**Before correction**	99.0	103.3	113.6	166.7	>200	>200
	**After correction**	98.9	102.1	105.4	95.8	89.8	91.0
	**SEER original estimate**	97.8	99.3	100.1	96.0	92.5	82.2
all cancers combined*	**Before correction**	67.7	66.8	73.9	117.2	>200	>200
	**After correction**	67.6	65.6	65.9	60.1	54.2	50.9
	**SEER original estimate**	64.8	60.9	58.5	55.5	51.8	44.3

*all cancers combined: all cancers excluding Non-Melanoma skin cancer (C00-C97 excl. C44).

## 3 Results

### 3.1 Application to German data and to SEER data

By applying the described method to German cancer registry data, we found 23,280 missed deaths for all cancers combined (**Table 4**). This corresponds to 0.9% of the patients who died. Intestinal cancer (C17 - C21, C26) was the diagnosis group with the highest number (4,141) of detected missed deaths. The highest proportions of missed deaths were detected for malignant melanoma (C43; 2.5% of deceased patients) and for thyroid and other endocrine glands (C73, C75; 1.9%). In contrast, the algorithm detected the lowest proportions of missed deaths for pancreas (C25; 0.4%) and for thorax (C33 - C34, C37 - C39, C45; 0.5%). Due to the stratification by diagnosis group, sex, age group and UICC stage the method was applied 349 times to the German data. In 260 of these strata, survival rates increased compared to the corresponding overall population, so that the analysis for missed deaths was carried out. The logistic regression was considered significant in 230 strata but led to more than 5% missed deaths (11 strata) or the proportion of deaths was below 10% (3 strata). Thus, logistic regression was applied 217 times and the scoring algorithm 43 times.

For SEER data, the method classified 696 patients as missed deaths, corresponding to 0.02% of deceased patients. Similar to the analysis of the German data, the proportions of detected missed deaths were the highest for thyroid and other endocrine glands (C73, C75; 0.7%) and malignant melanoma (C43; 0.3%). For the other diagnosis groups, survivors classified as missed deaths made up less than 0.1% of deceased persons. In total, SEER data was divided into 334 strata, with missed death analysis carried out in 74 strata (logistic regression 69 times, scoring 5 time).

Without excluding missed deaths in German data, estimated age-standardized relative survival for all cancer combined^1^ began to increase between 10 and 15 years of follow-up and exceeded 200% of survival in the corresponding population by the 25th follow-up year (**Table 7**). For malignant melanoma (C43) and prostate cancer (C61) this increase in estimated relative survival started between 5 and 10 follow-up years. After excluding the detected missed deaths, estimated survival decreased or only increased marginally over the entire follow-up time.

**Table 7:** Age-standardized relative survival rates [%] after excluding missed deaths and in brackets age-standardized relative survival [%] including missed deaths, estimated from German data and from SEER data, by diagnosis group, sex and follow-up time.

Diagnosis group	German data						SEER data					
	follow-up years						follow-up years					
	5	10	15	20	25	30	5	10	15	20	25	30
**Intestine (C17 - C21, C26)**	66.0 (66.1)	63.4 (64.2)	63.7 (74.6)	51.4 (150.1)	45.6 (>200)	29.1 (>200)	63.8 (63.8)	58.1 (58.1)	55.6 (55.6)	52.0 (52.2)	47.6 (48.4)	38.7 (38.8)
**Pancreas (C25)**	12.7 (12.8)	10.7 (11.3)	7.9 (15.3)	7.7 (48.9)	0.9 (>200)	0.0 (32.2)	9.9 (9.9)	6.8 (6.9)	5.3 (5.6)	3.9 (4.4)	2.5 (2.8)	1.8 (1.9)
**Thorax (C33 - C34, C37 - C39, C45)**	19.8 (19.8)	16.0 (16.6)	13.7 (20.2)	10.4 (57.9)	10.0 (>200)	12.7 (>200)	19.6 (19.6)	13.6 (13.6)	10.2 (10.2)	7.6 (7.6)	5.9 (6.2)	4.2 (4.2)
**Malignant Melanoma (C43)**	94.1 (94.3)	96.7 (99.1)	99.6 (119.8)	79.5 (>200)	59.1 (>200)	37.8 (>200)	89.6 (89.6)	88.5 (88.5)	89.1 (89.4)	89.2 (90.2)	80.0 (82.1)	71.9 (79.2)
**Breast (C50)**	87.4 (87.4)	83.5 (83.9)	84.4 (90.3)	75.3 (152.7)	63.6 (>200)	56.8 (>200)	90.9 (90.9)	86.7 (86.7)	83.4 (83.4)	79.4 (79.5)	74.9 (75.0)	59.3 (59.3)
**Prostate (C61)**	93.5 (93.5)	95.4 (96.0)	96.9 (109.1)	72.8 (>200)	64.8 (>200)	46.6 (>200)	97.8 (97.8)	99.3 (99.3)	100.1 (100.1)	96.0 (96.0)	92.5 (92.5)	82.2 (82.2)
all cancers combined^*^	63.6 (63.7)	61.1 (61.9)	60.7 (69.8)	51.4 (139.8)	45.9 (>200)	36.6 (>200)	64.8 (64.8)	60.8 (60.9)	58.4 (58.5)	55.1 (55.5)	50.7 (51.8)	42.5 (44.3)

*all cancers combined: all cancers excluding Non-Melanoma skin cancer (C00-C97 excl. C44).

Since there was only a small number of detected missed deaths in the SEER data, only small differences were observed between uncorrected and corrected age-standardized relative survival estimates. For all cancers combined^1^ the two survival rates differed after 30 follow-up years by 1.8 percent points. Among the diagnosis groups listed in **Table 7**, the largest correction (7.3 percent points after 30 years of follow-up) was made for malignant melanoma (C43).

### 3.2 Simulation results

On average over the six diagnosis groups tested, the algorithm detected 27% of simulated missed deaths at 1% simulated missed deaths, corresponding to 0.3% of all deaths (**Table 5**). With 2% and 5% simulated missed deaths, the algorithm determined that 0.7% and 2.2% of deaths were missed deaths, respectively. According to these results, the 0.9% missed deaths we detected in German data for all cancers combined would correspond roughly to 2.5% real missed deaths in the data. Within the diagnosis groups the highest proportions of missed deaths were detected for pancreatic cancer (C25) and the lowest proportions for breast (C50). In general, the algorithm detected higher proportions of missed deaths in cancers with poorer prognosis than in cancers with longer survival times. For pancreatic cancer (C25), the conditions for conducting the iterative search (**Figure 2**) were fulfilled in 85% of strata (based on sex, age group and historic stage A), whereas for breast cancer (C50), the algorithm kicked in for only 59% of the strata.

The sensitivity in **Table 5** is calculated as the proportion of simulated cases that are detected in relation to the total number of simulated cases. Across all diagnosis groups the sensitivity of the algorithm ranged between 27% and 38%, corresponding to 1% and 5% simulated missed deaths. Corresponding with the larger number of strata in which the algorithm was applied, sensitivity was higher for cancers with poor prognosis. The lowest sensitivity (14%) was observed for breast cancer (C50) and malignant melanoma (C43) at 1% simulated missed deaths and the highest sensitivity (47%) was seen for pancreatic cancer (C25) at 5% simulated deaths.

On average across simulations and across diagnosis groups, approximately 14% of classified missed deaths were actually not deceased patients. The proportion of false positive cases was smaller for cancers with poor prognosis than for cancer with long survival times. The highest proportion of false positive cases (52%) was for malignant melanoma (C43) with 1% simulated missed deaths, while only 4% of detected missed deaths were false positives for pancreatic cancers (C25) with 5% simulated missed deaths.

After introducing 5% missed deaths into the SEER data, the age-standardized 5-year relative survival rate for all cancers combined^1^ was 2.9 percent points higher than the original estimate and started to increase between 5 and 10 years of follow-up (**Table 6**). By diagnosis group, the increase in 5-year relative survival varied between 1.8 percent points for malignant melanoma (C43) and 6.0 percent points for pancreas (C25). Between 5 and 15 years of follow-up, the relative survival rates started to increase and exceeded 200% between 20 and 25 years of follow-up. After correcting for missed deaths, relative survival increased only slightly between 5 and 15 years of follow-up (for prostate (C61) and for malignant melanoma (C43)) and decreased otherwise. However, the corrected survival rates remained 2 to 7 percent points higher than the corresponding estimates without simulation over the entire follow-up time. In particular, the bias in the 5-year survival rates was reduced by only ≤ 0.5 percent points by the method.

## 4 Discussion

### 4.1 Achievements

We have developed a method for the classification of missed deaths in population-based cancer registry data in order to reduce bias in long-term survival estimates. When relative survival decreases substantially below 100% and subsequently begins to rise, the algorithm begins to identify exceptionally long-term and old survivors as likely to be misclassified and iteratively excludes these persons until relative survival no longer increases. In pooled German cancer registry data, the algorithm classified 23,280 supposed survivors as having died. Excluding these cases reduced the 30-year relative survival estimate for all cancers combined from over 200% to 36.6%. In SEER data, the algorithm classified only 696 “missed deaths”, reducing the 30-year survival estimate from 44.3% to 42.5%.

### 4.2 Focus on long-term survivors

The proportion of cases with missed deaths and the effect of these cases on survival estimates increases with follow-up time (1). Therefore, we focused on patients with long survival times and high age at the end of follow-up as candidates for missed deaths. With this approach, few missed deaths are detected if follow-up time is short, as demonstrated based on the simulation results: Among the 5% simulated missed deaths, nearly 20% had fewer than 5 years of follow-up. Only 0.2% of these cases were detected, although the algorithm detected about 38% of total simulated missed deaths (**Table 5**). Consequently, the 5-year relative survival for all cancers combined^1^ was corrected by the algorithm by only 0.1 percent points (**Table 6**). After 20 years of follow-up, however, relative survival was corrected from 117.2% to 60.1% by 57.1 percent points.

### 4.3 Associations with prognosis

The proportion of algorithm-classified missed deaths among deceased patients was relatively high for malignant melanoma (C43) compared to other cancer diagnoses, this was true in the German data as well as in the SEER data. Additionally, the simulations resulted in a high proportion of false positive algorithm-classified missed deaths and low sensitivity for this diagnosis (**Table 5**). This is likely due to the high proportion of melanoma survivors, even after a long follow-up time. In this case, the algorithm must exclude a large number of missed deaths to counteract increases in relative survival. For pancreatic cancer (C25) on the other hand, with a small proportion of long-term survivors, it is sufficient to exclude a far a smaller number of missed deaths to reduce the bias of the estimated survival rate. The sensitivity of the algorithm was about 20 percentage points higher for pancreatic cancer than for malignant melanoma, which also corresponded to lower false positive rates.

### 4.4 Migration between federal states as a possible source for missed deaths

In the German data, the number of algorithm-classified missed deaths is higher than in the SEER data. In addition to the disadvantages of passive follow-up practices used in German registries, which may not meet SEER follow-up standards (15), one reason for this difference may be the lack of a national death registry in Germany. In Germany, cancer registries as well as population registries are organized at the state level. If a patient moves to another federal state after cancer diagnosis, the original cancer registry cannot link the patient’s record to the death registry of another state. The original cancer registry may obtain the information that a person has moved to a different federal state, which can be used to censor the case, but the patient’s vital status is no longer available to the registry. In the past, the data transmitted from the cancer registries to the German Centre for Cancer Registry Data did not contain information about patient emigration, as these data were routinely collected only in some registries. Therefore, these cases could not be censored in survival analysis in the nationwide dataset. The Hamburg Cancer Registry provided information on emigration of cancer patients in the period from 1990 to 2014 to the Centre for Cancer Registry Data (**Table 8**). During this period, 3.2% of patients moved out of the catchment area of the cancer registry. Emigration was more prevalent among persons with cancers with good prognosis than among persons with more fatal cancers. Because the vital status is not updated for these patients, they are potential candidates for missed deaths in the German data. Since Hamburg is a city-state, emigration may occur less frequently in federal states with larger areas. However, it can be assumed that a substantial proportion of missed deaths in German cancer registry data was caused by emigration out of the catchment areas of the cancer registries.

**Table 8:** Number of emigrations out of the catchment area of the Hamburg cancer registry in the years from 1990 to 2014 by cancer site.

Diagnosis group	Emigrations	Persons
**Colorectum (C18 - C21)**	964 (3.1%)	31,057
**Pancreas (C25)**	36 (0.4%)	8,002
**Lung (C33- C34)**	319 (1.1%)	30,074
**female breast (C50)**	1,934 (5.4%)	35,550
**Prostate (C61)**	842 (3.3%)	25,241
**Total**	**4,095 (3.2%)**	**129,924**

### 4.5 Screening effects

The comparison of attained age and survival times between deceased and surviving persons at follow-up end assumes that both groups have similar survival times. This assumption can be incorrect if there are substantial advances in therapy or if, because of the introduction of screening, cancers are detected earlier over the course of follow-up (16, 17). Large improvements in survival raise the probability that patients who are still alive due to the new therapy or whose cancer has been detected early through screening will be misclassified as missed deaths. For this reason, the method tests at each iteration whether the proportion of detected missed deaths exceeds 5% of the total number of deaths. Above 5%, the iteration was canceled. The threshold was set to 5% of the total number of deaths after analyzing the expected proportion of missed deaths in the German cancer registry data. If the method is applied to data from other cancer registries with other expected proportions of missed deaths, this threshold should be adjusted.

### 4.6 Limitations and strengths

Bias in long-term survival estimates was reduced such that relative survival did not increase at the end of follow-up. The best results were achieved for cancers with high mortality, since survival rates differed strongly from those of the general population. For cancers with low mortality, however, the algorithm differentiated poorly between missed deaths and actual long-term survivors, as indicated by a high false positive rate in the simulation (**Table 5**, malignant melanoma (C43)). The method cannot be used to detect missed deaths with short follow-up time in relation to median survival time of the patients. In the simulation, the bias in short-term survival generated by missed deaths could be corrected only marginally (**Table 6**). The thresholds used for classifying missed deaths with logistic regression are obtained directly from the data. When the method is applied to cancer registry data with other survival times, these thresholds will be adapted automatically. Due to the stratification the algorithm is adjusted for prognostic factors such as stage or age.

## 5 Conclusion

The described method is a new approach for detecting missed deaths in data of population-based cancer registries. Surviving patients with extreme follow-up data (long follow-up time and old age) in relation to the corresponding data of deceased patients are classified as missed deaths. By applying the algorithm, long-term survival estimates can be corrected to such an extent that they do not exceed survival in the general population, thus especially increasing the validity of long-term survival or prevalence estimates. As the method has substantial limitations, for example regarding short-term survival, efforts for improving and ensuring high quality mortality data in cancer registries remain essential. The algorithm will be implemented in the R package ‘misseddeaths’ to be published on GitHub.

## Data availability statement

The German data that support the findings of this study are available on request at the German center for cancer registry data (www.Krebsdaten.de). The availability of these data is restricted due to legal reasons. The SEER data that were used in this study are available on request at Surveillance, Epidemiology, and End Results (www.seer.cancer.gov). Again, the availability of these data is restricted due to legal reasons. The simulated data that were used in the study were based on SEER data. Since only some dates of follow-up end were altered, the legal restriction for SEER data apply. These data are, however, available from the corresponding author upon reasonable request and with permission of Surveillance, Epidemiology, and End Results (www.seer.cancer.gov). Requests to access the datasets should be directed to German center for cancer registry data, www.Krebsdaten.de; Surveillance, Epidemiology, and End Results, www.seer.cancer.gov; SD, dahms@rki.de.

## Author contributions

The described method was developed by SD based on discussion with the two other authors. SD and BB wrote the manuscript with input from KK. The R package was created and designed by SD and BB. All authors read and approved the final manuscript.

## Footnotes

1 all cancers combined: all cancers excluding Non-Melanoma skin cancer (C00-C97 excl. C44).
